# Exploring barriers to using modern contraceptives and accessing safe abortion care in women who terminated unintended pregnancies in Southern Ethiopia

**DOI:** 10.1186/s12905-023-02793-3

**Published:** 2024-01-16

**Authors:** Mahlet A. Woldetsadik, Yeshihareg Yoseph, Mekonnen Degu

**Affiliations:** Family Guidance Association of Ethiopia, Hawassa, Ethiopia

**Keywords:** Abortion, Barriers to care, Ethiopia, Modern contraceptives, Pregnancy, Women

## Abstract

**Background:**

Unsafe abortion is a major medical and public health problem in Ethiopia and contributes significantly to abortion-related morbidity and mortality in the country. We explored women’s experiences with modern contraceptives and identified barriers to accessing safe abortion care and other sexual and reproductive health services.

**Methods:**

We recruited participants from six health clinics and a public hospital in southern Ethiopia. We conducted one-on-one interviews with 34 women aged 18–49 years who sought abortion care within the past twelve months of the study. Interviews were recorded, transcribed verbatim, translated to English, and imported into Dedoose software. We analysed the data using a directed content analysis.

**Results:**

Our findings show prevailing belief among participants that contraceptives caused infertility, abnormalities in subsequent pregnancies, and other side effects. Some of the women suggested that medical or unsafe abortions were a better alternative to using modern contraceptives for terminating unplanned pregnancies. Barriers to accessing safe abortion care included costs of services, lack of privacy, and fear of judgment from providers. Women who had negative experiences with providers were more likely to seek unsafe abortion the second time around.

**Conclusion:**

Providers need to address women’s concerns about using modern contraceptives, educate their clients about the best ways to prevent unwanted pregnancies, and provide non-judgemental services. In addition, comprehensive counselling services that include contraceptive counselling should be made available to women before, during, and after abortion care. Finally, providers should strive to be facilitators of, not barriers to, safe abortion care.

## Introduction

As of 2019, sub-Saharan Africa had the highest rate of abortion-related deaths in the world at 185 maternal deaths per 100,000 abortions [1]. It is estimated that over six million unsafe abortions occur in the region per year [1]. The World Health Organization (WHO) defines unsafe abortion as “a procedure for terminating an unwanted pregnancy either by persons lacking the necessary skills or in an environment lacking minimal medial standards or both” [2]. Unsafe abortion is a preventable but leading cause of maternal morbidities and mortality and can lead to permanent physical and mental health problems, as well as financial burdens for women, communities, and health systems [3]. For each woman that dies from complications, many more sustain permanent disabilities and become susceptible to an increased risk for long-term morbidities including various gynaecological injuries [4].

Unsafe abortion is a major medical and public health problem in Ethiopia and contributes significantly to maternal mortality in the country [5]. Although there has been a significant decrease in abortion-related mortality since the country reformed its penal code to enable women to obtain legal abortion of their own assertion that their pregnancy was a result of rape or incest [6], abortion-related morbidity in the country is still very high [5]. A national study estimated that 42% of unintended pregnancies in Ethiopia per year ended in abortion, out of which 73% were likely conducted in unsafe conditions [7]. Although the scaling up of legal abortion services throughout the health care system has improved since the legal reform, Ethiopian women continue to use unsafe abortion services outside of health facilities [8].

Family planning can prevent unwanted pregnancies and is a cost-effective way of reducing maternal mortality [9]. The contraceptive prevalence rate in Ethiopia among married women increased from 14% in 2005 to 41% in 2009 [10]. However, one in four married women in the country still have an unmet need for modern contraception, including oral hormonal pills, the intra-uterine device (IUD), the male condom, injectables, contraceptive implants, vaginal barrier methods, the female condom, birth control shot (Depo-Provera), and emergency contraception [11]. Access to contraceptives and safe abortion services have been shown to be the key factors that impact unsafe abortion rates, which are lowest in countries where contraceptive uptake is high [12]. Research has shown that the most effective way to prevent unintended pregnancies is through correct and consistent use of contraceptives. For instance, one study showed that each year, 260,000 to 272,000 maternal deaths could be averted worldwide by the use of contraceptives [13]. Limited or no access to contraceptives is one of the major causes leading to unwanted pregnancies in Ethiopia, where one in three pregnancies in the country are unintended [5].

Many quantitative studies have assessed the prevalence and risk factors associated with unsafe abortions in Ethiopia [7, 14]. However, there is still a lot to be learned about the context under which women choose to end pregnancies and their experiences with contraceptives prior to unintended pregnancies that are resolved by (unsafe) abortion. This qualitative study was designed to understand women’s experiences with regards to terminating unplanned pregnancies. Specifically, we gathered information about participants’ experiences with modern contraceptives and barriers to accessing and utilizing safe abortion services. In addition to gathering insights regarding obstacles and barriers, we asked participants for recommendations on how health institutions can encourage women to use sexual and reproductive health service, including safe abortion care.

## Methods

### Study setting and participant recruitment

We followed the COnsolidated criteria for REporting Qualitative research (COREQ) guidelines on the design and reporting of qualitative research [15]. We recruited participants from six health clinics and a public hospital providing sexual and reproductive health services from four towns in southern Ethiopia: Yirgalem, Sodo, Shashemene, and Hawassa. We used purposive sampling to recruit women who had sought abortion care in the last 12 months prior to the study and were between the ages of 18 to 49 years old. Local staff from each of the recruitment sites contacted eligible participants to explain the objective of the study and invited them to participate. We planned to continue recruitment until we reached saturation, a process by which the researcher continues to sample relevant cases until no new insights are being picked up from the data [16]. We reached saturation by the 34th interview. None of the invited individuals refused to participate. This study was approved by the Southern Nations, Nationalities, and Peoples’ Regional State Health Bureau’s Research Ethical Review Committee, in Hawassa, Ethiopia prior to recruitment and data collection (Ref.No.6–19/14/42). The Southern Nations, Nationalities, and Peoples’ Regional State Health Bureau’s Research Ethical Review Committee approved both written and oral informed consent to be sought from participants. All participants provided written or oral informed consent prior to enrolment in the study. All procedures performed in this study were in accordance with the ethical standards of the national research committee and with the 1964 Helsinki declaration and its later amendments or comparable ethical standards.

### Procedure

After obtaining basic demographic information from participants, we used a semi-structured interview guide to explore topics around participants’ experiences with modern contraceptives, terminating unwanted pregnancies and barriers to using modern contraceptives, and accessing safe abortion care. We also asked participants about the kind of information they would want about family planning, contraception methods, and safe abortion services. The interview guides were piloted with two staff from the Family Guidance Association of Ethiopia and revised before implementation. MAW who speaks Amharic fluently, along with two research assistants who spoke Woliyetegna and Oromiffa, conducted in-person interviews at a location chosen by the interviewee where confidentiality and privacy could be ensured. The research team did not know any of the participants prior to their enrollment in the study. Interviews lasted between 45 and 65 min. Prior to each interview, we told participants about the objectives of the study and emphasized that participation was completely voluntary. We reassured participants that the information they shared would be confidential and that they would not be identified by name in any publication. All participants provided oral or written consent. We conducted 28 interviews in Amharic, four in Woliyetegna and two in Oromiffa. We referred participants who requested additional counselling to the appropriate support services. All participants received transportation reimbursements. Three participants declined to be audio-recorded. All recordings were deleted at the end of the study.

### Data analysis

All recordings were transcribed verbatim, translated to English by MAW and uploaded into Dedoose software [17]. To ensure the quality of translations, YY back-translated a random selection of five English transcripts to Amharic. We used a directed content analysis approach to analyse the data, which began with the topics identified from the interviews as a guide to develop a structured codebook, while also allowing for flexibility so that additional themes could emerge directly from the data [18]. Two team members independently coded five transcripts, resolved coding and interpretation differences and further revised the codebook to ensure reliability of coding across interviews. MAW single-coded the rest of the interviews. To avoid potential researcher bias, the final codes were discussed with the entire research team, which also met regularly to identify and discuss emerging themes. We used paraphrases of the participants’ narratives in the body text and provided example illustrative quotes for barriers to using modern contraceptives and accessing safe abortion services in Tables 1 and 2, respectively.

**Table 1:** Women’s perceptions of factors associated with non-use of modern contraceptives in Southern Ethiopia

	(N = 34)		
**Perception**	**n**	**%**	**Examples**
Negative attitudes towards contraceptives; side-effects outweigh benefits	23	67	“I have heard that taking depo [Depo-Provera] for an unmarried woman is dangerous and can make her sterile. The nurse also told me that taking depo long term will cause fertility problems and it is better to use one of the long-term contraceptives at one time.” [22 years-old, employed]
			“I believe they [modern contraceptives] are dangerous. I had so many side effects including heart burn, fatigue, weight gain and I worry that they might also cause long term problems such as infertility, so I prefer to not use any.” [ 22 years-old, employed]
			“In our community, there is a widely held belief that if a woman takes contraceptives before getting married, she will be infertile. So, most women don’t use any type of modern contraception.” [23 years-old, student]
Preference for traditional contraceptive, mainly periodic abstinence	12	35	“I used to use my periods to regulate safe times to have intercourse. But I still got pregnant. Women have to be educated about this and other methods.” [24-years-old, unemployed]
			“My brother-in-law who is a doctor told me not to use it [modern contraceptives] so I only use my period to regulate the timing for safe sex.” [19-years-old, employed]
Experience with failed modern contraception	12	35	“I used an emergency [morning-after] pill within 72 h, but it didn’t have the instruction package when he [her boyfriend] bought it, so I don’t know if I followed the proper instructions.” [19-years-old, student]
			“I was using depo consistently and was shocked to find out that I had gotten pregnant. I am a mother of 5 children and cannot afford to feed another mouth. I am very sad and I feel like I am being forced to get an abortion because I was using something that I was told would prevent a pregnancy. I cannot have another child…” [35-years-old, housewife]
Lack of knowledge about modern contraceptives and the various options available	18	53	“I did not know much about contraception or abortion pills before I came here. The doctor gave me a phone number and told me that if I ever need abortion pills again, I could call the number and find a person who would sell it to me.” [19-years-old student]
			“I did not know anything about any sort of contraceptive before she [community worker] persuaded me to come to the clinic, I got tested for HIV and started using depo [Depo-Provera]” [25-years-old, employed]

**Table 2:** Women’s perception of barriers to accessing safe abortion services in Southern Ethiopia

	(N = 34)		
**Perception**	**n**	**%**	**Examples**
Cost of safe abortion services served as a deterrent	21	62	“Most women in this community are poor and it is not so easy for them to find 300 to 400 Birr for safe abortion.” [ 27 years-old, employed]
			“The first abortion I had was unsafe. I couldn’t afford the abortion so I went to the lady [individual without the requisite skills to provide safe abortion services] but I knew the service was provided in healthcare centers, I just couldn’t afford it.” [25-years-old, employed]
			“Women seek unsafe abortion because the cost is lower. Even 50 Birr might be too expensive for some women.” [22-years-old, employed]
Lack of privacy or confidentiality prevented women from going to health centers	9	26	“Women don’t come to health facilities to get an abortion because they are scared and don’t want to be seen. This is a small town and you don’t want to be seen entering SRH [sexual and reproductive health] clinics.” 27 years-old, employed]
			“When I went to get a test at the clinic, there were other people around the doctor and I was too shy to tell him what my problem was. So, I told him that I had diarrhea.” [19 years-old, student]
			“Women are afraid of being seen by community members, it’s a small town and people talk. There’s no confidentiality maintained by the providers.” [23-years-old, unemployed]
Fear of mistreatment by providers	9	26	“I was so scared to go and talk to the doctor at the health center about my problem. When I told him [the doctor], he insulted me. He called me a dog. But you know, I did not mind all of that abuse as long as he gave me the information on where to get my problem solved and he did. That’s all that mattered to me.” [18-years-old, student]
			“I was married at the time and living with HIV. My husband and I had decided that we did not want any children. I accidently got pregnant and went to a hospital to end the pregnancy. They [doctor and nurse] told me that they had used a surgical method or something they call the vacuum to clean my uterus and even showed me an ultra sound after the procedure. But after 4 months, I felt something move. At that time, my husband was bedridden and I did not have any sexual intercourse. I went back to the hospital and the same nurse and doctor told me that I was still pregnant and it seemed like the initial procedure did not ‘go through’. When I asked them how this could have happened, they said that maybe this baby was supposed to be born. So, I ended up having my daughter.” [25-years-old, employed]
			“In healthcare centers, there is a lot of mistreatment of women so women avoid going there to begin with even when they know that they should seek safe abortion services from there [health centers]” [25-years-old, employed]
Lack of information, health centers don’t advertise abortion services	7	21	“I got a pregnancy test at a health facility that provided safe abortion services, but I did not know about that until recently.” [18-years-old, employed]
			“We [she and her pregnant friends] don’t come to the clinic because they [health centers] don’t advertise the service of unsafe abortion.” [18-years-old, student]
			“Health care centers, including the one at the University, don’t give you enough information, you don’t feel free to talk to them and ask them about abortion.” [19-years-old, student]

## Results

We interviewed a total of 34 participants between May and June 2013 about their experiences with modern contraceptives and seeking and accessing abortion services. Twenty-seven (79%) participants were recruited from non-governmental sexual and reproductive health clinics, while the rest (*n* = 7) were recruited from a public hospital. More than 70% (*n* = 24) of participants were under 24 years-old, 44% (*n* = 15) were employed, and 70% (*n* = 24) were either married or in a relationship. Over 76% (*n* = 26) of participants had at least some secondary education, with 26% (*n* = 9) having attended some college (Table 3).

**Table 3:** Characteristics of women recruited from six non-governmental health clinics and a public hospital in southern Ethiopia (N = 34)

Characteristics	n	%
**Age (years)**		
18–20	11	32.4
20–24	13	38.2
25–29	7	20.6
30–34	1	2.9
35–39	2	5.9
**Occupation**		
Housewife	4	11.8
Student	11	32.3
Employed	15	44.1
Unemployed	4	11.8
**Marital Status**		
Married	8	23.5
In a relationship	16	47.1
Single	9	26.5
Divorced	1	2.9
**Education**		
Unable to read or write	2	5.9
Enrolled (high/middle)	6	17.6
Middle/high school drop out	17	50.0
Some college/university	9	26.5
**Setting**		
Non-governmental sexual and reproductive health clinics	27	79.4
Public hospital	7	20.6

All participants discussed a number of issues that prevented them from using modern contraceptives, led to unwanted pregnancies, and their decision to seek safe or unsafe abortion. Themes fell into two main categories: factors associated with contraceptive non-use, and personal and institutional attitudes that served as barriers to accessing safe abortion services. Participants also identified various entry points for improving uptake of contraceptives and access to safe abortion services.

### Barriers to using modern contraceptives

When asked about their experiences with using contraceptives, 44% (*n* = 15) of participants said they did not use any form of contraceptives, including traditional contraceptive methods such as periodic abstinence or withdrawal. Around 35% (*n* = 12) were using some sort of family planning including modern contraceptives, while the rest 21% (*n* = 7) were using only traditional contraceptive methods – mainly period abstinence, a natural family planning approach that entails abstaining from sexual intercourse during a woman’s fertile days.

Participants identified four main barriers to using modern contraceptives: negative attitudes towards contraceptives and perceived side-effects, preference for using traditional contraceptive methods, personal experiences with failed contraceptives, and lack of knowledge about the different types of contraceptive available to women. There was a prevailing belief among participants that side-effects of modern contraceptives outweighed their benefits. More than half of the participants said they were told by women in their community or health providers that modern contraceptives caused infertility, could damage or ‘thin’ the uterus, and result in a range of reproductive health problems. This issue was especially a major point of contention among most unmarried women (59%, *n* = 20) who said they hoped to have children in the future and did not want to risk having fertility problems. These women said they preferred to use traditional contraception methods and get an abortion in case they got pregnant. On the other hand, women who were using contraceptives such as pills or Depo-Provera prior to their accidental pregnancies had reservations about the effectiveness of these methods. Women who said they preferred to create a natural birth control method by regulating their menstrual cycles said they felt the information they had on how to use that method was outdated or incomplete. Table 1 summarizes perceptions of factors associated with non-use of contraceptives.

### Barriers to accessing safe abortion care

Participants discussed a range of personal and institutional issues that served as barriers to accessing safe abortion services. When asked about factors that might have made it difficult for women to access safe abortion services, participants identified four main barriers: being unable to afford the cost of safe abortion services, concerns about lack of privacy or confidentiality at health institutions, fear of judgment or mistreatment by providers, and lack of knowledge that health facilities provide safe abortion services. For most women in our study, the cost of abortion services at health institutions was high and a major barrier to seeking abortion from these centers.

Lack of privacy during visits was another concern for participants. Participants shared that in some cases, other people come in and out of the room as a doctor was talking to them, which hindered them from sharing their problems openly. Moreover, in small cities and towns where most of the women lived (71%, *n* = 24), there was usually one sexual and reproductive health center for the area. This was a major factor that prevented some women (26%, *n* = 9) from seeking services from health centers since they were either likely to know the providers working at the centers or did not want to be seen going into these facilities. In conjunction with lack of privacy, fear of mistreatment by providers was another factor that deterred women from seeking abortion services from health facilities. 26% of participants (*n* = 9) reported some form of mistreatment from providers, including being reprimanded for getting pregnant out of wedlock or wanting to ‘commit a sin’ – referring to women’s desire to terminate a pregnancy. Around one-quarter of participants mentioned that they had no idea the health centers they frequented for other family planning services also provided safe abortion care. Participants were reluctant to ask about these services and said they wished the centers advertised them openly. They also added that they hoped providers would share information about safe abortion services during conversations about family planning services. Table 2 contains illustrative quotes of participant perceptions of barriers to accessing safe abortion care.

### Recommendations for improving access to care

All participants offered at least one suggestion for improving the uptake of modern contraceptives and access to safe abortion services. Over 47% (*n* = 16) of women said health professionals had a responsibility to be well informed themselves and also educate their clients about the pros and cons of modern contraceptives. More than half of the participants (*n* = 20) called for reducing the cost of abortion services and enhanced pre- and post-abortion counselling on how to avoid unplanned pregnancies. Women who said they preferred using traditional methods said it would be helpful to learn more about how to use ovulation calendars and their menstrual cycles to avoid unwanted pregnancies. Other suggestions included addressing the attitudes of providers through targeted training and sensitivity programs. It was especially important to participants (*n* = 14) that they be informed about the pros and cons of each contraceptive method offered to them before they are asked to decide.

## Discussion

Increasing access to modern contraceptives has been shown to be one of the most effective ways of reducing maternal deaths in low-income countries [13]. Many countries have implemented programs designed to raise awareness and promote the use of modern contraceptives among their population. However, in most sub-Saharan African countries, including Ethiopia, the use of modern contraceptives is still very low, and unsafe abortion is a major factor contributing to maternal mortality and morbidity [6]. Our study explored the various factors that prevented women living in southern Ethiopia from using modern contraceptives and accessing safe abortion services.

Prior studies have identified a range of concerns women living in other low-income countries have about modern contraceptives including lack of knowledge, risk misperceptions, and negative social norms around premarital sexual activity [1, 19]. We found evidence of similar issues among our participants, where some women even suggested that medical or unsafe abortions were a better alternative to taking modern contraceptives for terminating unplanned pregnancies. This is a major concern for health providers and policy makers in the country, given that using abortion as a birth control method has major physical and psychological consequences [20]. The level of morbidity in the aftermath of unsafe abortions is high and can lead to short and long-term consequences, including abortion related deaths, chronic inflammation of the reproductive tract, and secondary infertility [21]. Moreover, post-abortion care from unsafe abortions costs the Ethiopian health system between $6.5 to $8.9 million dollars annually [22].

Studies have shown that some of the greatest barriers to getting safe abortion services are related to costs and stigma [23]. We found evidence of similar issues among the participants in our study, where women said they chose unsafe abortion services because they were unable to afford the cost of safe abortion care in health facilities. Participants also raised concerns about the need for comprehensive counselling sessions, which was not provided to them during their visits for abortion care. This absence of counselling services has various clinical implications. First, health facilities might simply not be equipped to provide counselling services either through lack of resources or counsellors. Providing contraception as part of post-abortion care is vital for preventing future unintended pregnancies and unsafe abortions [1]. Second, although family planning providers are expected to counsel women on various sexual and reproductive health issues, they might not have the appropriate training to counsel women seeking abortion services. Some participants in our study described that they did not feel comfortable talking to providers about safe abortion care and those who did said they felt judgment from providers. This problem could stem from providers’ unwillingness to provide abortion related services due to religious or personal attitudes towards terminating pregnancies. One way to mitigate this problem would be to support programs that attract prospective abortion care providers, which has been found to be effective in other similar settings [24].

A few study limitations should be noted. Our data were collected from women who sought abortion services from health facilities. As such, their concerns may underrepresent the magnitude and scope of barriers facing women who don’t come to health facilities and end their unwanted pregnancies through unsafe procedures. Since participants were recruited using purposive sampling, the themes identified from the narratives presented in this paper cannot be generalized to the larger population of women who terminate unintended pregnancies. Furthermore, these data were collected from only a sub-set of private and public facilities that provide comprehensive abortion care. Therefore, it is unclear to what extent the same issues would be identified among women who seek services from facilities not included in our study. However, we expect institutional cultures and capacity between the sites we sampled and others in the area to remain qualitatively similar. Finally, more research is needed to explore the challenges faced by women who end unintended pregnancies outside health facilities.

## Conclusion

Although Ethiopia has made commendable progress in improving access to modern contraceptives and safe abortion services, challenges still remain. Our findings suggest that improving women’s knowledge about modern contraceptives and addressing their concerns may be an important entry point for decreasing unintended pregnancies that might result in unsafe abortion. The issues regarding cost of safe abortion services and role of providers in influencing whether women take contraceptives or seek safe abortion services are of significant concern. These factors suggest a need to re-examine the cost of services and to ensure that providers are facilitators of, not barriers to, safe abortion care.

## Data Availability

The data presented in this article are not readily available because of confidentiality agreements with participants and research ethics board restrictions. Any reasonable requests should be directed to the corresponding author.
